# Impact of Different Post-Curing Temperatures on Mechanical and Physical Properties of Waste-Modified Polymer Composites

**DOI:** 10.3390/ma17215301

**Published:** 2024-10-31

**Authors:** Bernardeta Dębska, Bruna Silva Almada, Guilherme Jorge Brigolini Silva

**Affiliations:** 1 Faculty of Civil and Environmental Engineering and Architecture, Rzeszow University of Technology, Poznańska 2, 35-084 Rzeszow, Poland; 2 Departamento de Engenharia Civil, Universidade Federal de Ouro Preto, Campus Morro do Cruzeiro, Ouro Preto 35400-000, Brazil; bruna.almada@aluno.ufop.edu.br (B.S.A.); guilhermebrigolini@ufop.edu.br (G.J.B.S.)

**Keywords:** polymer composites, mechanical properties, post-curing temperature, ANOVA, utility profile, waste materials

## Abstract

One of the key trends affecting the future of the construction industry is the issue of ecology; therefore, current activities in construction aim to reduce the use of raw materials, which is made possible by including recycled materials in composites, among other methods. This article describes the results of tests conducted using four types of epoxy composites, i.e., composites modified with waste rubber (WR), composites modified with waste polyethylene (PE) agglomerate, glycolysate obtained using polyethylene terephthalate (PET) waste, and control unmodified mortars (CUM). Selected properties of the mortars were monitored during their maturation under laboratory conditions, as well as after post-curing at elevated temperatures in the range of 60 °C–180 °C. With the increase in the reheating temperature, an increase in the flexural strength of all types of mortars was noted, with the highest more than twofold stronger than the unmodified composites. The compressive strength increased up to a temperature of 140 °C, and then decreased slightly. The highest value of 139.8 MPa was obtained using PET mortars. Post-curing also led to a slight loss of mass of all samples in the range of 0 to 0.06%. Statistical methods were employed, which made it possible to determine the post-curing temperature and composite composition for which the determined properties are simultaneously the most beneficial, especially for the prefabricated elements.

## 1. Introduction

The future of construction is determined by important factors such as ecology and the development of modern material techniques. On the one hand, this is associated with the intensification of research and development work, and on the other hand, with optimization through the reduction of the amount of waste and energy consumption associated with the production and transport of building materials. The development of a closed cycle in the construction sector can contribute significantly to solving the problem of global shortages of natural resources and reducing the huge amount of waste produced by humans, which pollutes the environment. Environmental problems specifically affect the production, use, and consumption of plastics and rubber [1,2].

Plastics are materials that do not decompose into natural substances, and under long-term exposure to seawater and sunlight, they disintegrate into microscopic fragments, increasing their toxicity. Among the plastic waste produced in the world, the largest share is made up of polyolefins: polyethylene (PE), polypropylene (PP), and polyethylene terephthalate (PET). A significant amount of this waste comes from packaging materials, including PE bags and sacks, or PET bottles, and packaging, which, because of their popularity, are very difficult to dispose of, occupying huge volumes in landfills. Research related to the possibility of managing this type of plastic waste is of great importance in this context [3,4].

Due to the dynamic development of motorization and the improvement in quality of life, the purchase of cars is growing rapidly, which is why the excess of tire waste has become a global problem. Tire waste rubber is not biodegradable, and the cross-linked structure and various types of additives used in the production process mean that the natural decomposition process takes a long time, on the order of hundreds of years. The methods used to dispose of tire waste, such as incineration or storage, cause the formation of harmful gases, as well as serious soil, water, and air pollution [5,6,7,8,9,10,11].

There is a need to look for alternative methods of recycling, e.g., introducing waste into the composition of composites [12]. A composite that perfectly fits these criteria is resin concrete, classified as a group of concrete-like polymer composites. The basic composition of this material comprises synthetic resin (most often polyester, epoxy, polyurethane, or methacrylic), hardener, and aggregate, e.g., sand–gravel mixture and quartz flour, basalt or granite grit. In addition, various types of modifiers can be used, which should have a positive effect on the selected properties of polymer concrete [5,13,14]. The characteristic technical features of resin concrete include high mechanical strength, low shrinkage, very low water absorption, and high tightness, low abrasion, and exceptionally low roughness, i.e., the possibility of obtaining very smooth surfaces, excellent resistance to aggressive chemicals, and excellent adhesion to many other materials. The short setting time and the rapid increase in strength necessary to achieve assembly and operational efficiency are also important [15].

The production of resin composites using plastic or rubber waste is not only possible but also brings technical, economic, and environmental benefits, as demonstrated in the referenced publications [1,7,9,12,16], among others. However, in the case of polymer composites, it is very important to properly select the composition and parameters of the technological process (including temperature and maturation time) to obtain the most advantageous properties of the material in the context of the planned application [17]. For prefabricated polymer concrete elements, for technological reasons, it is advantageous to initially harden them at a low temperature, which promotes polymerization with the formation of a linear structure, and then to anneal the products for final cross-linking. Hardening at room temperature takes several hours, after which the reaction is not yet completely accomplished. The hardened composition achieves full mechanical, thermal, and chemical resistance only after several days. In order to maximize the final properties, additional heating (post-curing) is desirable [18]. In most cases, this procedure allows the composite elements to achieve the highest possible strength and become stable, but prolonged exposure to high temperatures can cause the resin matrix to decompose, similar to the aging phenomenon of composites in natural exploitation [19]. In this context, at the technological process design stage, the extent to which the resin matrix can be subjected to secondary curing without the risk of the deterioration of its thermal properties, as well as which mechanical and physical property values can be obtained as a result of the prolonged exposure of the composites to elevated temperatures should be examined [20]. It should also be taken into account that various types of fillers, including those obtained from recycling, can be used to change the properties of resin composites [21,22].

These issues were the inspiration for the research described in this paper, which is also a continuation of the research conducted for epoxy-bonded mortars, modified in both the resin matrix and the filler, with recycled materials, including PET, polyethylene, and rubber. This approach will allow for a comprehensive assessment of newly designed resin composites. The use of the above-mentioned waste significantly affects the properties of the composites obtained, which were described in previous work. The structure and properties of the material also depend, to a large extent, on the chemical structure of the hardener [14,23,24]. In turn, the curing time, i.e., the time until the epoxy groups react as completely as possible and the optimal properties of the material are obtained, depends not only on the type and amount of the hardener, but also on the composition of the composite and the curing temperature. The initial forming temperature and the rate of its increase are also very important [14,25]. The results of the studies described in this article indicate how the strength properties and the mass of the mortar samples change when heated at temperatures of 60, 80, 100, 140, and 180 °C, respectively, while including a given type of waste in the composition of the composites. This approach is a novel aspect and can be a valuable addition to the source literature database. Studying changes in the properties of composites cured at different temperatures makes it possible to select the most favorable curing conditions. However, those conditions for which the most favorable values are obtained, e.g., flexural strength, do not always lead to other optimal properties, such as the maximum temperature of thermal deformation. Thus, for the purpose of this work, statistical methods were used to search for technological parameters for which it is possible to simultaneously obtain the most favorable of all properties relevant to a particular application.

## 2. Materials and Methods

### 2.1. Materials

The subject of the study was resin mortars, the standard composition of which included the following: (1) resin binder (RB), (2) hardener, and (3) standard aggregate (SA).

(1) The RB in the CUM type mortars consisted of the Epidian 5 epoxy resin (SARZYNA CHEMICAL SP Z O.O., Nowa Sarzyna, Poland), while in the PET, PE, and WR mortars, it was a mixture of 91% by weight of Epidian 5 and 9% by weight of glycolysate obtained by heating the propylene glycol and polyethylene terephthalate (obtained from waste beverage bottles) for 1 h at a temperature of 85 °C.
(2) The hardener was triethylenetetramine (Z-1, SARZYNA CHEMICAL SP Z O.O., Nowa Sarzyna, Poland) in the amount of 10% by weight relative to the weight of the epoxy resin.
(3) The SA was standard sand, with a silica content of 98% and a grain size of 0 to 2 mm. In the PE and WR mortars, it was replaced by waste materials, i.e., polyethylene agglomerate from waste plastic bags and rubber granulate obtained from waste car tires, respectively, in a 10% volume on a fraction-to-fraction basis.

The selected properties of the resin and hardener used in the tests, developed on the basis of the manufacturer’s data [26], are presented in Table 1.

**Figure 1:**
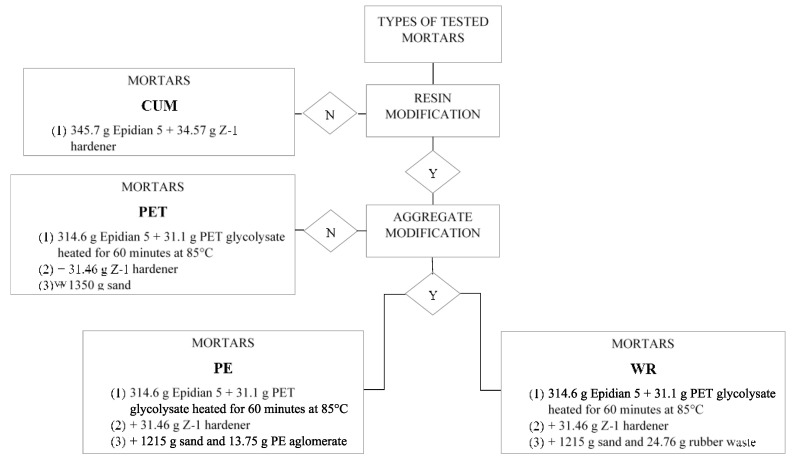
Diagram of process for obtaining individual types of mortars.

A constant volume ratio of the binder to the aggregate was assumed—RB/SA = 0.58. Four series of mortars were obtained according to the scheme shown in Figure 1.

### 2.2. Methods

The individual components of the mortar were dosed into a metal bowl and mixed in a laboratory mixer for 3 min, maintaining a constant mixer speed. The mortar prepared in this way was placed in steel molds with dimensions of 40 × 40 × 160 mm and mechanically compacted. The molds with mortar prepared in this way were stored in laboratory conditions at a temperature of 20 °C for 24 h and then demolded. After the samples were demolded, the individual series of mortars were weighed. One series of each type of mortar was held at a temperature of 20 °C, and the remaining samples were heated for 3 h in a laboratory dryer at temperatures of 60, 80, 100, 140, and 180 °C, respectively. Each sample was then weighed again and subjected to strength tests. The flexural and compression strength was determined using strength machines equipped with appropriate test inserts. The flexural strength was tested at an assumed load increase of 0.25 mm/min using a testing machine with a pressure of 150 kN (QC-505B1 COMETECH TESTING MACHINES CO., LTD Taiwan) for three samples for each type of mortar and heating temperature. The compressive strength was determined on each half of the sample obtained after this test. A 1500 kN hydraulic press (C6/4 MATEST, Arcore, Italy) was used, with an assumed load increase of 2.4 kN/s. Strength tests were carried out according to the requirements of the standard [27]. The research results were analyzed using the ANOVA module available in Statistica 13.

Analysis of variance (ANOVA) belongs to a group of statistical methods used to study observations that depend on one or more simultaneously acting factors. ANOVA explains the probability with which the isolated factors may be the reason for the differences between the observed group means. In this work, the influence of two factors was considered together, which is why a multifactorial analysis of variance was used.

Thanks to the options located on the Profile tab of the ANOVA/MANOVA module, it was possible to calculate and display the utility profile for the combination of both factors.

To identify potential chemical alterations in mortars after post-curing, the CUM and PET resin samples and the post-curing temperatures of 60 °C and 180 °C were selected, given the pronounced discrepancies observed in the mechanical and mass loss tests (see Section 3.1). The samples were ground (<45 μm), and pressed KBr pellets (5 tons) were produced, with a concentration of 1% for each sample, and these samples were evaluated using the Thermo-Fisher Scientific Nicolet iS5 FTIR transmission spectrometer (Thermo-Fisher Scientific, Waltham, MA, USA). The spectra were obtained within the wavenumber of 4000–400 cm^−1^, with a resolution of 4 cm^−1^ and 32 scans per sample.

The microstructural characterization of the resin samples was conducted using a scanning electron microscope (SEM) (VEGA 3, TESCAN, Brno, Czechia) equipped with a secondary electron (SE) detector. The samples were mounted on stubs with carbon adhesive tape and subsequently coated with a thin layer of gold using a QUORUM Q150T sputter coater (QUORUM, Lewes, UK). Micrographs were acquired in the SE mode to visualize the resin and its aggregates.

## 3. Results and Discussion

### 3.1. Mechanical and Physical Properties

The experimental results are summarized on a spreadsheet from the Statistica 13 program. For each type of mortar, the mean values and standard deviations of each feature tested were calculated, taking into account the post-curing temperature (Table 2).

Based on Table 2, it can be concluded that the application of post-curing the samples at different temperatures has an effect on the strength properties and the change in the mass of the mortars. Increasing the curing temperature increases the mobility of the molecules, facilitating the further reaction of the epoxy groups with the hardener and increasing the cross-linking density. In general, mechanical properties increase with increasing temperature, but at 180 °C, a decrease in compressive strength is already observed. Increasing the post-curing temperature also resulted in a decrease in the mass of the mortar samples tested, which may be caused by the degradation of the resin matrix.

**Figure 2:**
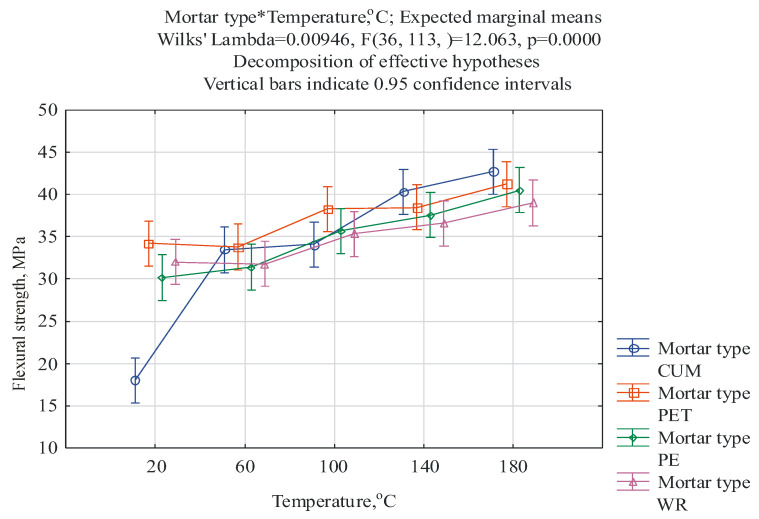
Graphical interpretation of the effect of interactions between the mortar type and post-curing temperature obtained for flexural strength.

**Figure 3:**
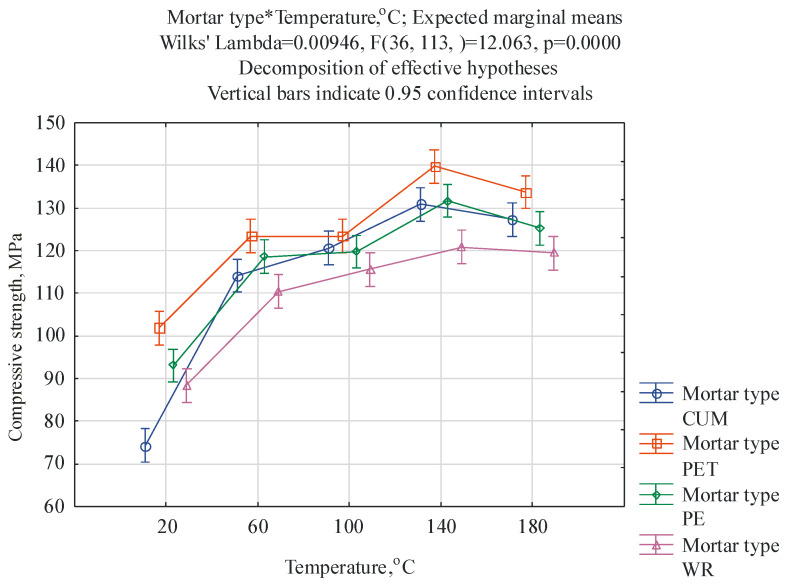
Graphical interpretation of the effect of the interaction between mortar type and post-curing temperature obtained for compressive strength.

**Figure 4:**
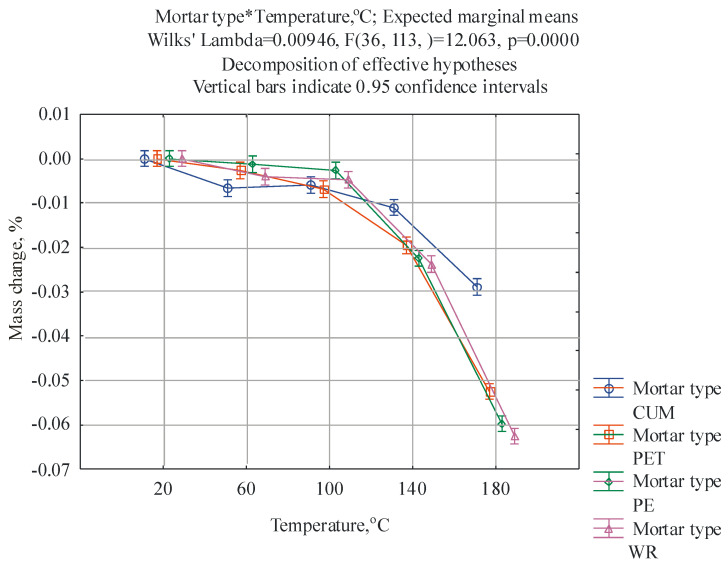
Graphical interpretation of the effect of the interaction between mortar type and curing temperature obtained for mass change.

For the results obtained, a multifactorial analysis of variance was performed in relation to the following two factors: (1) the type of mortar and (2) the post-curing temperature. The ANOVA/MANOVA module available in the Statistica 13 program was employed. The first factor occurs at four levels, i.e., CUM, PET, PE, and WR, while the second factor at occurs at five levels, i.e., 20 °C, 60 °C, 100 °C, 140 °C, and 180 °C. The purpose of this analysis was to check whether the type of mortar and post-curing temperature have a statistically significant effect on the mechanical properties and mass change of the mortar samples and whether there is an interaction between these factors. The F-test was applied, with the level of statistical significance of α = 0.05 and a confidence level of 95%. The results of the ANOVA test are given in Table 3. The results of the two-way analysis of variance, given in Table 3, indicate that both the type of mortar and the post-curing temperature significantly differentiate the flexural and compressive strength and the change in mass because the related *p*-values are lower than α = 0.05. There is also a significant interaction, which means that the effect of a given post-curing temperature depends on the type of mortar. The significance of these interactions is confirmed by the interaction graphs generated in the Statistica 13 program, presented in Figure 2, Figure 3 and Figure 4.

It is clearly visible that the pattern of average flexural strengths for individual types of mortars, post-cured at a given temperature, is similar. However, it turns out that there is a particularly large difference between the flexural strength characteristic of the uncured control unmodified mortar (CUM) and all the others (the bars indicating the standard errors of the means do not overlap). At a post-curing temperature of 180 °C, the flexural strength of all types of mortars still increases, but in this case, the highest of 42.7 MPa is characteristic of the control unmodified mortars. For comparison, Elalaoui et al. [19] noted that at a lower resin content (13 wt.%), the increase in flexural strength is noticeable up to a temperature of 150 °C, and at 200 °C, a decrease in this property is observed. Jin et al. [14] also observed a positive effect of the post-curing process on the flexural strength of epoxy concretes, but concluded their tests at a maximum temperature of 60 °C. Aruniit et al. [18] found that the flexural strength increased from 40 MPa to 52 MPa when the material was cured at 60 °C for 6 h, while curing at a temperature higher than 60 °C did not cause a significant increase in the flexural strength of the tested material.

Similarly to flexural strength, the compressive strength of mortars cured at laboratory temperature is clearly different for control unmodified mortars and other mortars, with CUM mortars having the lowest compressive strength at this temperature, equal to 74.3 MPa, and PET mortars the highest (101.9 MPa). With the increase in the post-curing temperature, the highest strength of the PET mortars is maintained, while for CUM mortars, this property increases significantly and remains at a level comparable to the compressive strength of the PE mortars up to a temperature of 180 °C. In the temperature range of 60 °C–80 °C, the lowest (although still very high in the range of 110.5–119.4 MPa) compressive strength values are characteristic of WR mortars. For all types of mortars tested, a decrease in compressive strength was noted for the temperature of 180 °C compared to those obtained for the temperature of 140 °C. Similar dependencies are characteristic of the concretes described in Ref. [19], for which the reduction in compressive strength occurred between temperatures of 100 °C and 150 °C. In turn, the authors of the article [25] noticed that with the resin content of 12 wt.%, the compressive strength of the composites increased significantly, even at a temperature of 200 °C. El-Hawary and Abdel-Fattah [25] also noted that the strength properties of the tested resin concretes strongly depended on the type of epoxy resin used.

Initially (60 °C), the highest values of mass change were characteristic of unmodified mortars; however, with the increase in the post-curing temperature to 140 °C and 180 °C, this trend was reversed, and the mass of the modified samples changed more significantly. It should be emphasized that for all types of mortars, the mass loss is small, even at a temperature of 180 °C; the highest, equal to 0.063%, was noted for the WR type mortars.

**Figure 5:**
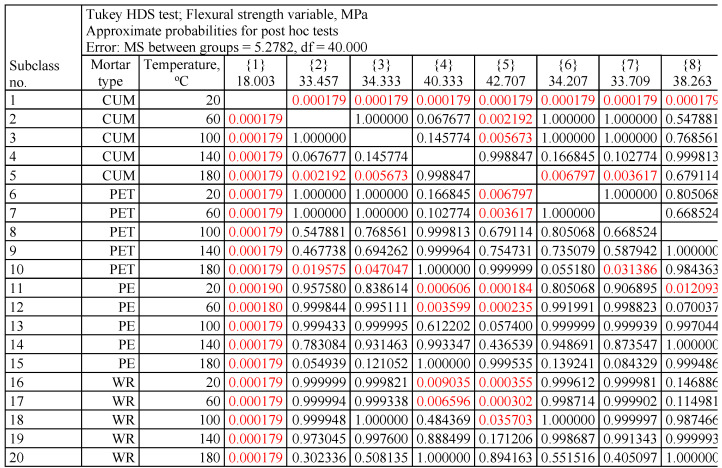
Fragment of the Tukey test results sheet obtained for flexural strength (statistically significant differences (*p* < 0.05) are marked in red).

To assess the detailed differences, a post hoc test was performed. Due to the relatively small numbers, the Tukey HSD test was selected, based on grouping means. An example of a portion of the results table for this flexural strength test is presented in Figure 5.

Due to the large size of the resulting tables, detailed results of the Tukey HSD test are shown in the Supplementary Materials (Figures S1–S3). The results of the Tukey test confirm the relationships shown in Figure 2, Figure 3 and Figure 4 in detail. The most statistically significant differences (*p* < 0.05, marked in red in Figures S1–S3) occur in the case of mass change, with fewer for compressive strength, and the least for flexural strength. In the case of flexural strength, the largest statistically significant differences occur between the control mortar cured at 20 °C and all other cases of mortars and temperatures.

The quality of a prefabricated polymer element is influenced by many factors. This quality can be expressed through the values of the physicochemical properties, among others, taking into account the fact that, depending on the planned application of the element, different features may be important. It is essential to select the composition and conditions for obtaining the material in a given application in such a way that all the key features for this application are at a satisfactory level. Searching for these relationships is possible using the Profile option, also available in the ANOVA/MANOVA module of the Statistica 13 program. For each of the properties tested, a response utility profile was defined, i.e., values from the range [0, 1] were assigned, indicating satisfaction with obtaining a result at such a level. For the strength parameters, the rule was that the higher the intensity of this feature, the better. In turn, when the mass is changed, it is optimal when its values are close to zero.

**Figure 6:**
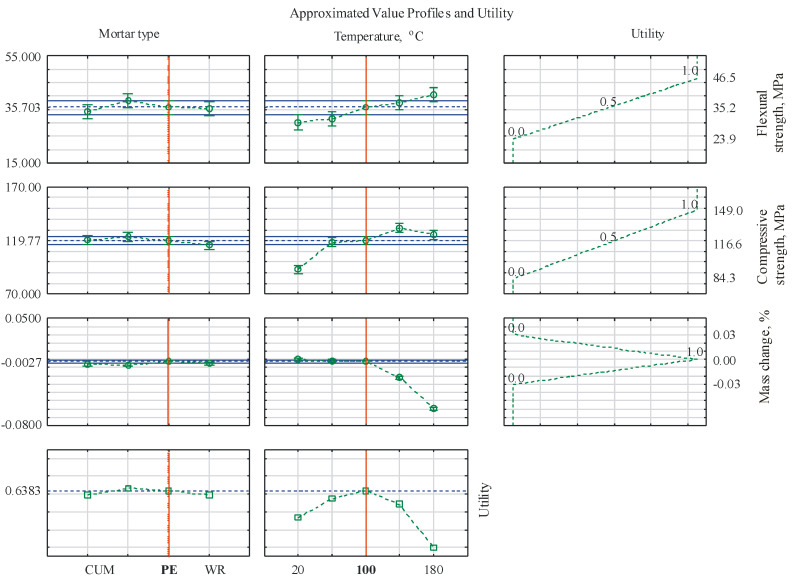
Approximated value profiles and utility.

The flexural strength was assigned values from the following range: 23.9 MPa—0 (undesirable), 32.5 MPa—0.5 (intermediate), and 46.5 MPa—1.0 (desirable). Similarly, for the compressive strength, the following range was assigned: 84.2 MPa—0 (undesirable), 116.6 MPa—0.5 (intermediate), and 149 MPa—1.0 (desirable). In turn, the mass change should be small; therefore, the following range was adopted: −0.03%—0 (undesirable), 0%—1 (desirable), and 0.03%—0 (undesirable) (Figure 6).

After the analysis, it can be concluded that the most advantageous features of epoxy mortars can be obtained by using PET and PE modification and by post-curing the product at a temperature of 100 °C. The total utility for such a system is high, at 63.8%.

### 3.2. FTIR and SEM

**Figure 7:**
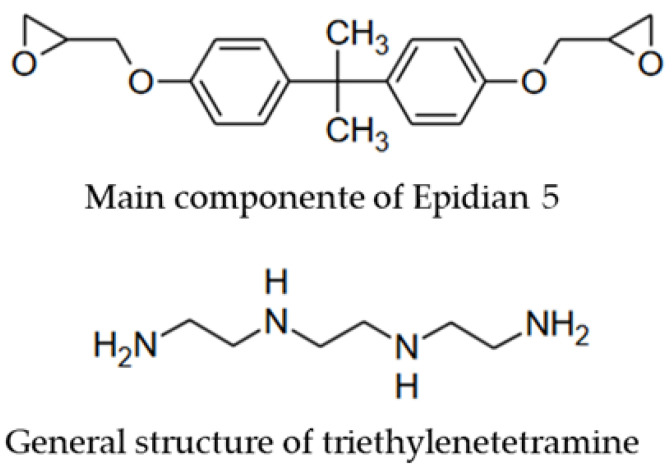
Epidian 5 and triethylenetetramine structures [29].

Figure 7 illustrates the structure of the Epidian 5 resin and the triethylenetetramine hardener, which were used in this investigation. As stated by Escola et al. [28], the oxirane rings present in Epidian 5 resin act as sites for crosslinking reactions. Consequently, in an FTIR spectrum, this functional group (800–1000 cm^−1^) is no longer present when interacting with multifunctional amine-type hardeners, such as triethylenetetramine, and hydroxyl functional groups (3300–3700 cm^−1^) are generated [28,29]. Moreover, the application of temperature during the curing process influences the hardening rate. The presence of residual absorption bands in the region of approximately 920 cm^−1^ has been observed in samples cured at low temperatures [30].

**Figure 8:**
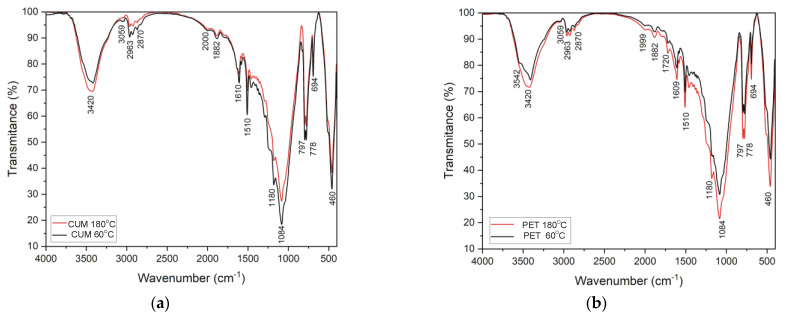
FTIR spectra of (**a**) CUM samples and (**b**) PET samples.

The fundamental vibration modes of the different organic groups observed in the FTIR spectrum of the CUM and PET samples, cured at 60 °C and 180 °C (Figure 8), allowed for the determination of the existing functional groups, as indicated in Table 4. The spectra of the CUM and PET samples are similar, in general, since the mortar with PET-modified resin also contains, for the most part, Epidian 5 resin. However, an absorption band at ~1720 cm^−1^ was identified at 1720 cm^−1^ in the PET samples at 60 °C and 180 °C. This band is indicative of the C=O stretch vibration, which is present in the glycolyzed product of PET, along with aromatic C-H bands at 1456–1504 cm^−1^ and alkyl C-H at ~2870 cm^−1^ [31,32,33].

In all samples, the presence of characteristic hydroxyl group absorption bands (3000 cm^−1^ to 3600 cm^−1^) was observed, suggesting that hardening occurred in both the Epidian 5 (CUM) reference resin and the (PET) modified resin. The present study was unable to identify the disappearance of the characteristic vibration bands of the oxirane rings because of the presence of the aggregate, which is composed mainly of quartz. The mineral in question displays spectra with characteristic Si–O–Si vibrations at 800 cm^−1^ and 1085 cm^−1^, which overlap on the peaks of the oxirane rings [31].

Furthermore, the spectra demonstrated that there was no translation of peaks or formation of new functional groups with increasing temperature. However, a band deformation at approximately 3540 cm^−1^ was observed in the samples at 60 °C, with a more pronounced deformation evident in the sample with PET-modified resin. This band is associated with the stretching vibration frequency of O-H and N-H of triethylenetetramine [31,34]. It is important to note that the O-H band resulting from the cross-linking reaction overlaps on the corresponding band originating from the traces of moisture present in the reagents [30]. Despite this overlap, this result may indicate that the utilization of elevated post-curing temperatures may potentially influence the structural configuration of the hardened resin and/or the rate of hardening, which could explain the observed strength gain in the mechanical results. However, further analysis is required to confirm this hypothesis. In order to gain further insight, the authors propose the implementation of additional microstructural assessments, including differential scanning calorimetry (DSC), transmission electron microscopy (TEM), and Raman spectroscopy.

**Figure 9:**
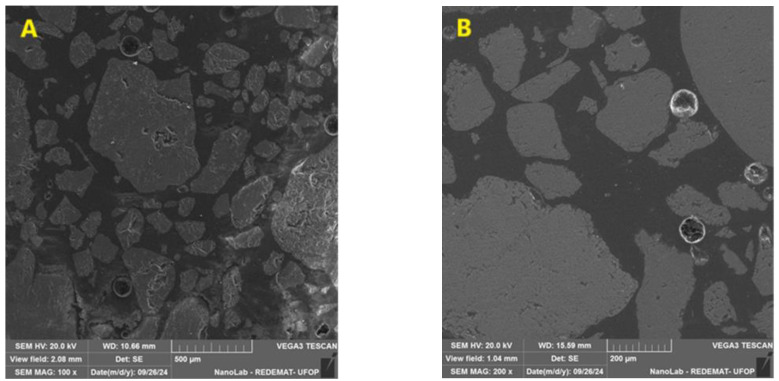

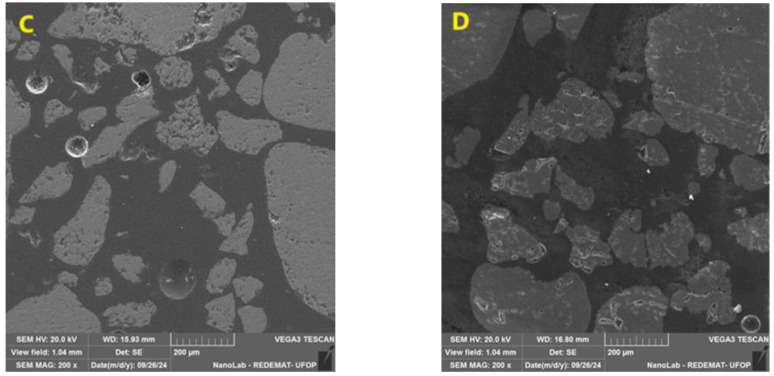
SEM images of samples: (**A**) CUM cured at 60 °C; (**B**) CUM cured at 180 °C; (**C**) PET cured at 60 °C, and (**D**) PET cured at 180 °C.

Figure 9 presents SEM images of CUM and PET samples, cured at 60 °C and 180 °C. No microstructural features were observed in the resin or at the resin–aggregate interface that could explain the differences in compressive strength between the samples. This suggests that the variation in mechanical performance is likely due to factors beyond the visible microstructural characteristics captured in this analysis.

## 4. Conclusions

The nature of the structure of the hardened resin composite depends on the structure of the resin and the hardener and their content, as well as on the temperature and hardening time. Additional heating of the elements after removal from the mold allows for the removal of internal stresses created during curing and has an impact on the formation of the physical and mechanical properties of the composite. The results of the tests conducted can be summarized as follows:

- The mechanical properties increase with an increase in heating temperature, but at 180 °C, a decrease in compressive strength is already observed.
- The effect of post-curing the samples on their flexural strength is most visible in the case of the control unmodified mortars, which at 20 °C, were characterized by a strength of 18 MPa, which was only 50% of the strength of the modified mortars, while at 180 °C, the CUM-type mortars achieved the highest flexural strength of 42.7 MPa.
- The increase in the post-curing temperature resulted in a small decrease (from 0% to 0.063%) in the mass of the mortar samples tested, which may be caused by the beginning of the degradation process of the resin matrix in the zone near the surface.
- Based on the response utility profile defined for each property, it was determined that the most advantageous features of epoxy mortars can be obtained by employing simultaneous modification with PET glycolysate and PE waste agglomerate and by post-curing the product to a temperature of 100 °C. The total utility of such a system is high, at 63.8%.

## Figures and Tables

**Table 1:** Selected properties of Epidian 5 resin and Z-1 hardener.

Properties	Epidian 5	Z-1
Characteristics	Basic unmodified bisphenol A-based liquid resin	Aliphatic amine-based hardener
Epoxy number, mol/100 g	0.480–0.515	-
Epoxy equivalent, g/eq	194–208	-
Viscosity (25 °C), mPas	20,000–30,000	-
Color, Gardner/Hazen	max. 2/–	-
Amine number, mg KOH/g	-	Min. 1100
Density (20 °C), g/cm^3^	-	0.978–0.983

**Table 2:** Mean values of the tested parameters, along with the standard deviation obtained for individual mortar types and hardening temperatures.

Mortar Type	Temperature, °C	Flexural Strength, MPa ± Std. Dev.	Compressive Strength, MPa ± Std. Dev.	Mass Change, % ± Std. Dev.
CUM	20	18.0 ± 0.3	74.3 ± 7.2	0.000 ± 0.000
	60	33.5 ± 3.6	114.2 ± 4.9	−0.007 ± 0.002
	100	34.1 ± 3.9	120.6 ± 1.1	−0.006 ± 0.002
	140	40.3 ± 1.2	130.9 ± 1.7	−0.011 ± 0.003
	180	42.7 ± 2.6	127.3 ± 1.8	−0.029 ± 0.001
	General	33.7 ± 9.2	113.5 ± 21.4	−0.011 ± 0.010
PET	20	34.2 ± 2.3	101.9 ± 3.1	0.000 ± 0.000
	60	33.8 ± 2.3	123.4 ± 1.5	−0.003 ± 0.001
	100	38.3 ± 0.9	123.4 ± 4.5	−0.007 ± 0.001
	140	38.5 ± 1.8	139.8 ± 2.2	−0.019 ± 0.002
	180	41.2 ± 1.5	133.8 ± 2.2	−0.052 ± 0.002
	General	37.2 ± 3.3	124.5 ± 13.6	−0.016 ± 0.020
PE	20	30.2 ± 3.8	93.1 ± 2.1	0.000 ± 0.000
	60	31.4 ± 1.4	118.6 ± 0.9	−0.001 ± 0.001
	100	35.7 ± 2.7	119.8 ± 1.6	−0.003 ± 0.003
	140	37.6 ± 2.9	131.7 ± 0.6	−0.022 ± 0.001
	180	40.5 ± 1.1	125.3 ± 7.2	−0.059 ± 0.002
	General	35.1 ± 4.5	117.7 ± 13.9	−0.017 ± 0.024
WR	20	32.0 ± 1.8	88.4 ± 0.8	0.000 ± 0.000
	60	31.8 ± 0.7	110.5 ± 2.6	−0.004 ± 0.000
	100	35.4 ± 1.3	115.6 ± 1.8	−0.005 ± 0.001
	140	36.6 ± 2.6	120.9 ± 1.3	−0.024 ± 0.002
	180	39.0 ± 2.8	119.4 ± 5.3	−0.063 ± 0.000
	General	34.9 ± 3.3	110.9 ± 12.5	−0.019 ± 0.024

**Table 3:** ANOVA results for mechanical properties and mass changes of specimens, including mortar type and post-curing temperature, parameterization with sigma restrictions, and decomposition of effective hypotheses.

Properties	Effect	df	SS	MS	F	*p*
Flexural strength, MPa	Mortar type	3	93.87	31.29	5.93	0.002
	Temperature, °C	4	1104.72	276.18	52.32	0.000
	Mortar type*Temperature, °C	12	463.86	38.66	7.32	0.000
Compressive strength, MPa	Mortar type	3	1521.5	523.8	46.73	0.000
	Temperature, °C	4	574.9	3143.7	280.43	0.000
	Mortar type*Temperature, °C	12	857.6	71.5	6.37	0.000
Mass change, %	Mortar type	3	0.0006	0.0002	81.82	0.000
	Temperature, °C	4	0.0211	0.0053	2120.38	0.000
	Mortar type*Temperature, °C	12	0.0019	0.0002	62.33	0.000

df: degree of freedom; SS: sum of squares; MS: mean square; F: test statistics; *p*: probability.

**Table 4:** Assignments of the IR bands to vibrational modes of atomic groups.

Wavenumber (cm^−1^)				Functional Group
CUM 60 °C	CUM 180 °C	PET 60 °C	PET 180 °C	
3419	3424	3542, 3410	3424	O-H/N-H stretch
3057, 3037	3060, 3037	3059, 3037	3059, 3037	C-H
2964	2963	2963	2963	CH_3_ stretch
2927, 2870	2920, 2870	2926, 2870	2928, 2870	CH_2_ stretch
1783	1785	1785	1785	C=O
		1719	1720	C=O stretch
1609, 1581, 1510, 1459, 1361	1613, 1581, 1510, 1458, 1361	1610, 1581, 1458, 1510, 1362	1609, 1581, 1510, 1459, 1361	Aromatic ring mode/N–H bend
1411	1413	1412	1410	NH_2_ scissors
1383	1383	1383	1383	C-CH_3_
1293	1290	1296	1295	C–C–C stretch
1247	1248	1247	1247	C–C–O stretch
1180	1179	1179	1180	Si–O–Si stretch
1083, 693	1084, 694	1083, 694	1084, 694	C–H out of plane bend
797	796	796	797	Si–O–Si stretch
778	778	778	778	C–H out of plane bend
460	460	460	460	Si–O–Si bend

## Data Availability

The original contributions presented in the study are included in the article/Supplementary Material, further inquiries can be directed to the corresponding author.

## Supplementary Materials

The following supporting information can be downloaded at: https://www.mdpi.com/article/10.3390/ma17215301/s1, Figure S1: Tukey test result sheet obtained for flexural strength; Figure S2: Tukey test result sheet obtained for compressive strength; Figure S3: Tukey test result sheet obtained for mass change.
