# Supplementary material for: Impact of Different Post-Curing Temperatures on Mechanical and Physical Properties of Waste-Modified Polymer Composites
Source: Materials (Basel). 2024 Oct 31;17(21):5301. doi: 10.3390/ma17215301 (PMC11547642; doi:10.3390/ma17215301)
Supplement: Supplementary file 1 [file materials-17-05301-s001.zip › materials-3261970-supplementary.pdf]

Supplementary Materials

for

Impact of Different Post-Curing Temperature on Mechanical and Physical Properties of Waste Modified Polymer Composites

Subclass no.	Tukey HDS test; Flexural strength variable, MPa<br>Approximate probabilities for post hoc tests<br>Error: MS between groups = 5.2782, df = 40.000											
	Mortar type	Temperature, °C	{1}<br>18.003	{2}<br>33.457	{3}<br>34.333	{4}<br>40.333	{5}<br>42.707	{6}<br>34.207	{7}<br>33.709	{8}<br>38.263	{9}<br>38.487	{10}<br>41.240
1	CUM	20		0.000179	0.000179	0.000179	0.000179	0.000179	0.000179	0.000179	0.000179	0.000179
2	CUM	60	0.000179		1.000000	0.067677	0.002192	1.000000	1.000000	0.547881	0.467738	0.019575
3	CUM	100	0.000179	1.000000		0.145774	0.005673	1.000000	1.000000	0.768561	0.694262	0.047047
4	CUM	140	0.000179	0.067677	0.145774		0.998847	0.166845	0.102774	0.999813	0.999964	1.000000
5	CUM	180	0.000179	0.002192	0.005673	0.998847		0.006797	0.003617	0.679114	0.754731	0.999999
6	PET	20	0.000179	1.000000	1.000000	0.166845	0.006797		1.000000	0.805068	0.735079	0.055180
7	PET	60	0.000179	1.000000	1.000000	0.102774	0.003617	1.000000		0.668524	0.587942	0.031386
8	PET	100	0.000179	0.547881	0.768561	0.999813	0.679114	0.805068	0.668524		1.000000	0.984363
9	PET	140	0.000179	0.467738	0.694262	0.999964	0.754731	0.735079	0.587942	1.000000		0.993166
10	PET	180	0.000179	0.019575	0.047047	1.000000	0.999999	0.055180	0.031386	0.984363	0.993166	
11	PE	20	0.000190	0.957580	0.838614	0.000606	0.000184	0.805068	0.906895	0.012093	0.008684	0.000267
12	PE	60	0.000180	0.999844	0.995111	0.003599	0.000235	0.991991	0.998823	0.070037	0.052338	0.000964
13	PE	100	0.000179	0.999433	0.999995	0.612202	0.057400	0.999999	0.999939	0.997044	0.992303	0.305168
14	PE	140	0.000179	0.783084	0.931463	0.993347	0.436539	0.948691	0.873547	1.000000	1.000000	0.904298
15	PE	180	0.000179	0.054939	0.121052	1.000000	0.999535	0.139241	0.084329	0.999486	0.999879	1.000000
16	WR	20	0.000179	0.999999	0.999821	0.009035	0.000355	0.999612	0.999981	0.146886	0.113166	0.002303
17	WR	60	0.000179	0.999994	0.999338	0.006596	0.000302	0.998714	0.999902	0.114981	0.087544	0.001687
18	WR	100	0.000179	0.999948	1.000000	0.484369	0.035703	1.000000	0.999997	0.987466	0.973844	0.215318
19	WR	140	0.000179	0.973045	0.997600	0.888499	0.171206	0.998687	0.991343	0.999993	0.999952	0.609780
20	WR	180	0.000179	0.302336	0.508135	1.000000	0.894163	0.551516	0.405097	1.000000	1.000000	0.999465

Figure S1. Tukey test result sheet obtained for flexural strength.

Subclass no.	Mortar type	Temperature, °C	{1} 74.300	{2} 114.20	{3} 120.62	{4} 130.87	{5} 127.32	{6} 101.92	{7} 123.38	{8} 123.43	{9} 139.78	{10} 133.75
	Tukey HDS test; Compressive strength variable, MPa<br>Approximate probabilities for post hoc tests<br>Error: MS between groups = 11.210, df = 40.000											
1	CUM	20		0,000179	0,000179	0,000179	0,000179	0,000179	0,000179	0,000179	0,000179	0,000179
2	CUM	60	0.000179		0,693228	0,000222	0,003188	0,007571	0,136896	0,131555	0,000179	0,000180
3	CUM	100	0.000179	0,693228		0,055181	0,624026	0,000182	0,999946	0,999929	0,000181	0,003134
4	CUM	140	0.000179	0.000222	0.055181		0.998392	0.000179	0.431451	0.443201	0.168377	0.999901
5	CUM	180	0.000179	0.003188	0.624026	0.998392		0.000179	0.994535	0.995288	0.006264	0.689246
6	PET	20	0.000179	0.007571	0.000182	0.000179	0.000179		0.000179	0.000179	0.000179	0.000179
7	PET	60	0.000179	0.136896	0.999946	0.431451	0.994535	0.000179		1.000000	0.000239	0.049626
8	PET	100	0.000179	0.131555	0.999929	0.443201	0.995288	0.000179	1.000000		0.000243	0.051942
9	PET	140	0.000179	0.000179	0.000181	0.168377	0.006264	0.000179	0.000239	0.000243		0.780025
10	PET	180	0.000179	0.000180	0.003134	0.999901	0.689246	0.000179	0.049626	0.051942	0.780025	
11	PE	20	0.000182	0.000179	0.000179	0.000179	0.000179	0.179298	0.000179	0.000179	0.000179	0.000179
12	PE	60	0.000179	0.983283	0.999999	0.007442	0.190852	0.000223	0.957758	0.953762	0.000179	0.000468
13	PE	100	0.000179	0.868122	1.000000	0.024835	0.415993	0.000188	0.997980	0.997608	0.000180	0.001324
14	PE	140	0.000179	0.000193	0.024433	1.000000	0.981271	0.000179	0.253479	0.262213	0.308856	1.000000
15	PE	180	0.000179	0.026066	0.970307	0.859808	0.999999	0.000179	1.000000	1.000000	0.000791	0.228486
16	WR	20	0.001203	0.000179	0.000179	0.000179	0.000179	0.002077	0.000179	0.000179	0.000179	0.000179
17	WR	60	0.000179	0.996859	0.059481	0.000179	0.000212	0.223179	0.003855	0.003660	0.000179	0.000179
18	WR	100	0.000179	1.000000	0.938509	0.000426	0.013253	0.001816	0.360361	0.349729	0.000179	0.000185
19	WR	140	0.000179	0.628163	1.000000	0.070006	0.689246	0.000181	0.999988	0.999984	0.000181	0.004130
20	WR	180	0.000179	0.922109	1.000000	0.017021	0.332402	0.000194	0.993400	0.992432	0.000179	0.000925

Figure S2. Tukey test result sheet obtained for compressive strength.

Subclass no.	Tukey HDS test; Mass change variable, MPa<br>Approximate probabilities for post hoc tests<br>Error: MS between groups = 0.000, df = 40.000											
	Mortar type	Temperature, °C	{1}<br>0.0000	{2}<br>-0.0066	{3}<br>-0.0060	{4}<br>-0.0110	{5}<br>-0.0289	{6}<br>0.0000	{7}<br>-0.0026	{8}<br>-0.0069	{9}<br>-0.0196	{10}<br>-0.0525
1	CUM	20		0,001169	0,005168	0,000179	0,000179	1,000000	0,868908	0,000742	0,000179	0,000179
2	CUM	60	0.001169		1.000000	0.130512	0.000179	0.001169	0.220270	1.000000	0.000179	0.000179
3	CUM	100	0.005168	1.000000		0.036210	0.000179	0.005168	0.527011	1.000000	0.000179	0.000179
4	CUM	140	0.000179	0.130512	0.036210		0.000179	0.000179	0.000189	0.190862	0.000184	0.000179
5	CUM	180	0.000179	0.000179	0.000179	0.000179		0.000179	0.000179	0.000179	0.000180	0.000179
6	PET	20	1.000000	0.001169	0.005168	0.000179	0.000179		0.868908	0.000742	0.000179	0.000179
7	PET	60	0.868908	0.220270	0.527011	0.000189	0.000179	0.868908		0.152557	0.000179	0.000179
8	PET	100	0.000742	1.000000	1.000000	0.190862	0.000179	0.000742	0.152557		0.000179	0.000179
9	PET	140	0.000179	0.000179	0.000179	0.000184	0.000180	0.000179	0.000179	0.000179		0.000179
10	PET	180	0.000179	0.000179	0.000179	0.000179	0.000179	0.000179	0.000179	0.000179	0.000179	
11	PE	20	1.000000	0.001169	0.005168	0.000179	0.000179	1.000000	0.868908	0.000742	0.000179	0.000179
12	PE	60	0.999927	0.020641	0.080279	0.000179	0.000179	0.999927	0.999953	0.012609	0.000179	0.000179
13	PE	100	0.846601	0.241436	0.559619	0.000190	0.000179	0.846601	1.000000	0.168683	0.000179	0.000179
14	PE	140	0.000179	0.000179	0.000179	0.000179	0.001803	0.000179	0.000179	0.000179	0.748048	0.000179
15	PE	180	0.000179	0.000179	0.000179	0.000179	0.000179	0.000179	0.000179	0.000179	0.000179	0.000387
16	WR	20	1.000000	0.001169	0.005168	0.000179	0.000179	1.000000	0.868908	0.000742	0.000179	0.000179
17	WR	60	0.229025	0.859743	0.990036	0.000596	0.000179	0.229025	0.999873	0.763442	0.000179	0.000179
18	WR	100	0.069049	0.991001	0.999971	0.002484	0.000179	0.069049	0.980397	0.971784	0.000179	0.000179
19	WR	140	0.000179	0.000179	0.000179	0.000179	0.031894	0.000179	0.000179	0.000179	0.156362	0.000179
20	WR	180	0.000179	0.000179	0.000179	0.000179	0.000179	0.000179	0.000179	0.000179	0.000179	0.000179

Figure S3. Tukey test result sheet obtained for mass change.
